# HIV-1 Latency in Monocytes/Macrophages

**DOI:** 10.3390/v6041837

**Published:** 2014-04-22

**Authors:** Amit Kumar, Wasim Abbas, Georges Herbein

**Affiliations:** UPRES EA4266, SFR FED 4234, Pathogens and Inflammation Laboratory, Department of Virology, CHRU Besançon, University of Franche-Comte, F-25030 Besançon, France; E-Mails: amit.aiims2005@gmail.com (A.K.); wazim_cemb@hotmail.com (W.A.)

**Keywords:** HIV-1, latency, monocytes, macrophages, microglial cells

## Abstract

Human immunodeficiency virus type 1 (HIV-1) targets CD4^+^ T cells and cells of the monocyte/macrophage lineage. HIV pathogenesis is characterized by the depletion of T lymphocytes and by the presence of a population of cells in which latency has been established called the HIV-1 reservoir. Highly active antiretroviral therapy (HAART) has significantly improved the life of HIV-1 infected patients. However, complete eradication of HIV-1 from infected individuals is not possible without targeting latent sources of infection. HIV-1 establishes latent infection in resting CD4^+^ T cells and findings indicate that latency can also be established in the cells of monocyte/macrophage lineage. Monocyte/macrophage lineage includes among others, monocytes, macrophages and brain resident macrophages. These cells are relatively more resistant to apoptosis induced by HIV-1, thus are important stable hideouts of the virus. Much effort has been made in the direction of eliminating HIV-1 resting CD4^+^ T-cell reservoirs. However, it is impossible to achieve a cure for HIV-1 without considering these neglected latent reservoirs, the cells of monocyte/macrophage lineage. In this review we will describe our current understanding of the mechanism of latency in monocyte/macrophage lineage and how such cells can be specifically eliminated from the infected host.

## 1. Introduction

Since the discovery of human immunodeficiency virus type 1 (HIV-1) in 1983 till now, HIV-1 continues to be a major public health concern worldwide (UNAIDS, 2013). Realizing the devastating potential of HIV-1, the scientific community united to find solutions to fight this virus. Vigorous and desperate efforts resulted in the development of highly active antiretroviral therapy (HAART) in 1996 [1,2,3]. HAART has significantly improved and extended the lifespan of HIV-infected patients to a great extent [1]. Patients using HAART have HIV-1 levels below the detection limit of conventional techniques [2]. However, HAART is not able to completely eradicate HIV from infected individuals. Even interruption of HAART for few weeks usually results in detectable viremia in HIV-infected patients [3]. In-depth analysis of the problem revealed that HIV continues to exist in several cellular reservoirs due to poor drug penetration in anatomical viral sanctuaries, establishment of viral latency or cryptic viral replication [4,5]. Several novel carrier molecules that can reach these anatomical viral sanctuaries and deliver HAART are under development [6,7,8]. Significantly, HAART is able to inhibit the new infection of susceptible cells by the HIV virion [5,9]. Importantly, latency is established within few infected cells very early during acute infection [10,11]. HAART does not hamper the release of new viral progeny from those cells in which HIV has been integrated and latency has been already established [12]. Therefore, even if HAART is successfully delivered to the restricted viral zone, complete eradication is not possible without targeting the latent reservoirs.

Latency is defined as infection state in which no infectious viral particles are produced from the infected cells. However, this state is reversible upon stimulus [13]. Viral latency is one of the several mechanisms of viral persistence within the infected host [14,15,16]. Notably, viral latency is a rare event. There is an estimate that only in one cell out of 10^6^–10^7^ infected cells latency is established [11]. HIV-1 principally targets CD4^+^ T cells and cells of monocyte/macrophage lineage [17]. Upon infection, HIV reverse transcribes and integrates a copy of its genome into the host chromatin that further transcribes as cellular mRNA and governs HIV pathogenesis [18,19]. Infection of CD4^+^ T cells usually resulted in their lysis. However, some infected CD4^+^ T cells revert in order to become memory cells and reach the resting state [9,20]. These cells have a prolonged lifespan and thus permit the persistence of virus. The concept of HIV latency in resting CD4^+^ T cells is well investigated [5,9,16].

Like CD4^+^ T cells, monocyte/macrophage lineage cells are regarded as the early targets for HIV-1 infection [21,22]. These cells display both CD4 receptor as well as CCR5 or CXCR4 coreceptor on their surface [23,24]. Notably, cells of monocyte/macrophage lineage are more resistant to the cytopathic effect of HIV. Thus they can harbor virus for longer periods of time [25]. Upon entry into tissues, monocytes irreversibly differentiate into macrophages and tissue resident macrophages such as microglial cells in the brain [25]. Since macrophages are present in most organ systems they therefore can disseminate the virus throughout the body. These characteristics classified the cells of monocyte/macrophage as important viral reservoirs. Viral reservoirs are the infected cell types that contain latent virus or low levels of ongoing replication [9,26]. In this review, we will focus on the monocyte/macrophage lineage cells and discuss critically the evidences describing latency and associated molecular mechanisms in them. Derived information could help in the development of novel therapeutic strategies against HIV-1.

## 2. General Mechanisms of Latency

In general, latency can be classified into two types, pre-integration latency and post-integration latency [5,16].

### 2.1. Pre-integration Latency

**Figure 1:**
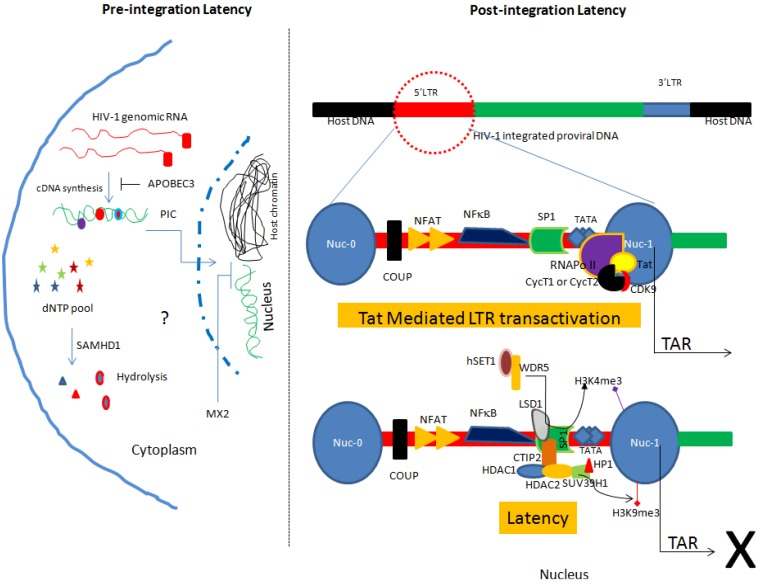
Model of pre-integration and post-integration latency in HIV-1 infected monocytes/macrophages. Pre-integration latency is governed by interplay of host restriction factors including APOBEC3, SAMHD1 and MX2. Such form of latency is more relevant in the cells of monocyte/macrophage lineage. On the other hand, post-integration latency is manifested by several mechanisms that include chromatin remodeling, epigenetic mechanisms and host-encoded miRNAs. Furthermore, Tat mediated reactivation has been also shown. PIC: Pre-Integration Complex.

Integration of HIV-1 proviral DNA into the host genome is a prerequisite for favorable viral pathogenesis. Upon HIV-1 entry into the cell, HIV-1 RNA is reverse transcribed into DNA and further assembled in the form of the pre-integration complex (PIC) [27] (Figure 1). The PIC is comprised of viral proteins (integrase, matrix, capsid, Vpr) and dsDNA. The PIC is later transported into the nucleus where it may integrate into the host genome. Pre-integration latency may be a consequence of poor reverse transcription efficiency and inhibition of nuclear transport of the PIC [18] (Figure 1). This form of latency has been well described in CD4^+^ T cells [28,29,30]. Data indicate that pre-integration latency is very common *in vivo* [31]. Furthermore, Pierson and colleagues reported that pre-integration latency decay is rapid in resting T cells [31].

On the other hand, macrophages can sustain large amounts of unintegrated HIV-1 DNA for longer periods of time (at least 30 days) [32]. Additionally, selective persistent viral gene expression and induction of chemokines e.g., CXCL9 and CXCL10 have been reported in macrophages harboring unintegrated viral DNA [32]. Taken together, data indicate that the unintegrated HIV-1 DNA in macrophages may significantly contribute to the viral pathogenesis in infected individuals. Importantly, there are only few reports where unintegrated viral DNA has been detected in macrophages derived from patient’s samples. For instance, in few studies [33,34] the presence of unintegrated HIV DNA has been detected in the brain of infected patients. However, concrete clinical evidence suggesting the role of unintegrated viral DNA in latency in patient derived macrophages is largely lacking and needs further investigation.

In addition, a limited dNTPs pool and several host restriction factors (RFs) pose a check on HIV-1 replication in the cells of monocyte/macrophage lineage and may contribute to the pre-integration latency. These RFs include APOBEC3 [35], sterile alpha motif (SAM) domain and HD domain-containing protein 1 (SAMHD1) [36] and the recently identified MX2 (myxovirus resistance 2, also called MXB) [37]. APOBEC3 plays an important role in triggering G to A hypermutation of HIV-1 genome (Figure 1). Notably, SAMHD1 reduces the dNTPs pool in macrophages by hydrolyzing dNTPs into their precursors (nucleosides and triphosphates) resulting in inefficient viral reverse transcription [36] (Figure 1). Additionally, MX2 has been shown to inhibit HIV replication in several susceptible cell types including macrophages [37]. Furthermore, MX2 inhibits HIV infection at post entry level by hindering the nuclear accumulation and integration of proviral DNA into the host chromatin [37].

Recently in a high throughput host restriction factor identification study, McKnight’s research group screened 19,121 human genes in HIV-infected cells using an siRNA library. They identified 114 genes which influence HIV infection to a significant extent [38]. Furthermore, they observed that inhibition of all members of the PAF1 family improves the efficiency of HIV reverse transcription and proviral DNA integration [38]. Notably, PAF-1 is also expressed in monocyte/macrophage lineage cells [36]. However, viral proteins including Vif [39] and Vpx [40] have been reported to target these restriction factors in the cells of monocyte/macrophage lineage. HIV-1 accessory protein Vif hampers the APOBEC3 mRNA synthesis and promotes the degradation of APOBEC3 protein by 26S proteasome [39,41]. Similarly Vpx triggers the proteasome-mediated degradation of SAMHD1 [40].

### 2.2. Post-integration Latency

As the name suggests, post-integration latency is established with the integration of HIV-1 proviral DNA into host chromatin followed by silencing of HIV-1 gene expression (Figure 1). Several mechanisms including epigenetic gene silencing [16], transcription gene silencing (TGS), and post transcriptional gene silencing have been described to explain establishment and maintenance of latency in the target cells (reviewed in [5,9,42]). The post-integration latency represents true latency responsible for the persistence and on activation, dissemination of HIV-1 [5]. Herein, we will briefly described post latency mechanisms and information available in context to the cells of monocyte/macrophage lineage.

#### 2.2.1. Site of Integration and Chromatin Remodeling

The host chromatin is organized into heterochromatin (densely packed, transcriptionally inactive) and euchromatin (loosely packed, transcriptionally active) regions [43]. Insertion of HIV-1 into the heterochromatin region can favorably support the latency. However, findings suggest that HIV-1 proviral DNA preferentially integrates into the euchromatin region [20,44,45]. Transcriptional interference has been suggested as one possible mechanism responsible for integrated proviral DNA expression suppression [46]. Notably, most of the data regarding HIV-1 latency and integration has been derived from CD4^+^ T cells. Like CD4^+^ T cells, Barr and colleagues investigated the integration site in primary macrophages. They sequenced 754 putative integration sites and found that in macrophages also HIV-1 preferentially integrates into the transcriptionally active region of the host chromatin [47]. Similar findings have also been reported from other laboratories [48,49]. The HIV-1 proviral integration sites in monocytes and microglial cells (CNS resident macrophages) have not yet been explored [49].

TGS is the most preferential mechanism of HIV-1 latency establishment [50]. However, whether such mechanisms also operate in the cells of monocyte/macrophage lineage is an area of ongoing research. It is well established that chromatin organization and reorganization influence the gene expression. HIV-1 proviral DNA also follows the same rules applied to the host genes. The role of histone H3 lysine 9 trimethylation (H3K9me3) in heterochromatin formation and transcriptional silencing of integrated HIV-1 has been described [51,52,53,54,55,56]. Notably, irrespective of the HIV-1 integration site in the host genome, two nucleosomes nuc-0 and nuc-1 interact with the HIV-1 promoter in a spatial manner [57] (Figure 1). In addition, HIV-1 gene transcription from proviral DNA is only possible with the displacement of nuc-1 [57]. The findings have been shown in U1 cells (a promonocytic cell line). These data collectively suggest that chromatin remodeling is a common mechanism of HIV-1 latency establishment and regulation in monocyte/macrophage lineage subsets.

#### 2.2.2. Involvement of Host Transcription Factors and Viral Proteins in Latency

Integrated HIV-1 proviral DNA is delimited at both ends by the long terminal repeats (LTR). The 5'LTR has binding sites for several transcription factors including the glucocorticoid receptor, COUP, USF, AP1, TCF-1alpha, C-Myc, SP1, CTF/NFE, NFAT and NF-kappaB [57] (Figure 1). These transcription factors can act in harmony to regulate the proviral DNA expression. For example, SP1 recruits C-Myc to the 5'LTR of proviral DNA that in turn recruits histone deacetylase (HDAC) 1. HDAC1 induces chromatin remodeling responsible for the suppression of HIV-1 gene expression [58]. In addition, C-promoter binding factor-1 (CBF-1), COUP-TF interacting protein 2 (CTIP2), YY1, and LSF have been also reported to be involved in the recruitment of HDACs to the proviral promoter and establishment of latency [25,59,60,61].

Similar findings have been reported in microglial cells where CTIP2 has been shown to recruit HDAC1 and HDAC2 to the 5'LTR of HIV-1 proviral DNA [53]. In addition, CTIP2 also interacts with SUV39H1, a methyl transferase responsible for H3K9me3 which subsequently promotes the recruitment of HP1 protein to the 5'LTR leading to the local heterochromatization and induction of the latency (Figure 1). Worth mentioning, Desplats and colleagues determined the expression of CTIP2 in the postmortem brain tissue of HIV^+^ control, latent HIV^+^ and HIV encephalitis (HIVE) cases. They found significantly higher amounts of CTIP2 in the latent HIV^+^ cases as compared to HIV^+^ controls and HIVE cases. Interestingly, using double labeling they detected the presence of CTIP2 in the microglial cells of latent HIV^+^ cases only. Their data further strengthen the role of CTIP2 in regulating latency in microglial cells [62]. In addition to the above-described factors, recently the role of the lysine-specific demethylase (LSD1) in regulating HIV gene expression in a synergistic manner with CTIP2 in microglial cells has been shown [63,64]. Furthermore, LSD1 helps in the recruitments of CTIP2 and hSET1/WDR5 (members of hCOMPASS complex) at the Sp-1 binding sites of the HIV proximal promoter resulting in increased H3K4 trimethylation (H3K4me3) [64] (Figure 1). In response to an increase in H3K4me3, viral gene expression is repressed. Moreover, upon reactivation release of LSD1 and hSET1/WDR5 from viral LTR and decrease in the H3K4me3 has been reported further confirming the role of these factors in modulating latency in microglia [63,64]. This feature of LSD1 seems to be specific for cells of monocyte/macrophage lineage. In contrast, there are reports describing the role of LSD1 as a transcriptional activator in latently infected CD4^+^ T cells [65]. Different roles of LSD1 in microglial cells suggest involvement of LSD1 as an anchor protein which helps in the recruitment of other factors at LTR [63,64].

Furthermore, Barber and colleagues reported that in the macaque brain, simian immunodeficiency virus latency is established by interplay of interferon-beta and dominant-negative isoforms of CCAAT/enhancer-binding protein-beta (C/EBP-beta) resulting in the inhibition of the histone acetylation and in suppression of LTR activation. Since microglial cells are long-lived latent reservoir of HIV-1 in infected individuals, targeting the factors involved in regulation of latency, especially CTIP2 could be one plausible therapeutic strategy [66,67].

Recently, multiple TCF-4 binding sites have been reported in 5'LTR of proviral DNA. Furthermore, formation of protein complexes which include TCF-4, beta-catenin and SMAR1, a nuclear matrix binding protein, at −143 positions on 5'LTR has been shown to repress proviral gene expression in astrocytes [68]. Notably, cells of monocyte/macrophage lineage have intact beta-catenin signaling [69]. Therefore, the role of these proteins in inducing latency in cells of monocyte/macrophage lineage is also speculated.

In addition to the cellular transcription factors, HIV-1 latency is also influenced by viral transactivator protein called Tat [70] (Figure 1). HIV-1 transcription can occur even in the absence of Tat, however, only prematurely terminated short transcripts are produced [70]. The positive transcription elongation factor (P-TEFb) favors the generation of complete transcript from host or proviral DNA [70,71]. P-TEFb is made up of a catalytic subunit (CDK9) and of a regulatory subunit (CycT1 or CycT2) [70] (Figure 1). Tat binds to the 5'LTR of proviral DNA and directs P-TEFb to the RNA polymerase II resulting in productive full-length transcription [70]. In addition, CDK2 phosphorylates Ser90 on CDK9 and assists in HIV-1 transcription [72]. Targeting CDK2 in regulating HIV-1 latency has been postulated [72,73].

In addition, recently Kashanchi’s research group reported the differential expression of two Baf proteins (Baf 53 and Baf170) in HIV-1 infected cells as compared to their uninfected counterpart. Notably, Baf proteins play an important role in chromatin remodeling. In addition, their data revealed that CycT1/CDK9 phosphorylates Baf53 in the presence of Tat resulting in activation of viral gene transcription [74].

Noteworthy, as compared to macrophages, monocytes have low level of CycT1; therefore have low functional P-TEFb molecules. Lack of P-TEFb can limit the transcription of HIV-1 in monocytes [75] that could be one possible mechanism of latency in the monocytes. However, exogenous expression of CycT1 does not contribute to the LTR driven transcription in them [75]. Interestingly, fusion of monocytes with HIV-1 permissive cell line (for example human embryonic kidney cell line) resulted in Tat mediated transactivation of LTR [75]. The findings raise the possibility of other potent cellular factor(s) which can regulate HIV-1 transcription [75]. Worth mentioning, CycT1 expression and phosphorylation level of CDK9 are increased in monocyte-derived macrophages (MDMs) [75].

In addition, in the context of transcription factors involved in the suppression of Tat-mediated LTR activation, OTK18, a zinger finger has been identified [76]. OTK18 is an antiretroviral transcription factor produced in macrophages upon HIV-1 infection. Notably, OTK18 expression has been found to be highly specific. For example, OTK18 is undetectable in microglial cells and found specifically in perivascular macrophages [76].

2.2.3. miRNA and HIV Latency

Besides regulating host gene expression, miRNAs also influences viral gene expression [77,78]. For example, for efficient replication in PBMCs and Hela cells, HIV-1 inhibits the expression of miRNA cluster miR-17/92 [79]. The role of this anti-miRNA cluster has been also demonstrated in monocytes and macrophages [79]. Notably, monocytes are less susceptible to HIV-1 infection than macrophages. Presence of higher levels of anti-HIV miRNA (miRNA-382, miRNA-223, miRNA-150 and miRNA-28) in monocytes than MDMs may contribute to lesser susceptibility of monocytes for HIV-1. In addition, suppression of these anti-HIV miRNAs in monocytes results in increased HIV replication. On the other hand, addition of miRNA mimics to the MDMs result in the decrease in HIV replication [80]. Strategically manipulating the miRNAs could provide a potent anti-HIV therapeutic tool.

## 3. Latency in Monocyte/Macrophage Lineage Subsets

### 3.1. Latency in Monocytes

Monocytes are the integral part of our immune system. Several initial research findings indicate the presence of HIV-1 DNA in monocytes isolated from infected patients [81]. Upon co-culturing monocytes derived from HIV-1 infected patients or latently infected THP-1 myeloid cell line with concanavalin A-activated T cells (isolated from healthy donors), the production of infectious HIV virions has been reported [82]. Detection of HIV-1 in monocytes of patients undergoing HAART further supports these findings [11,83]. Since circulating monocytes have a relatively short life span as compared to resting T cells (in which latency is well established), significance of latency in these cell types is not well understood. Whether it is a case of true latency or just a result of low level ongoing HIV-1 replication is not very clear [84,85,86]. However, from viral reservoir and virus dissemination into anatomical sanctuaries point of view, the role of HIV-1 infected monocytes cannot be ignored [87].

### 3.2. Latency in Macrophages

Macrophages are an important HIV reservoir as they have the capacity to harbor virus [88] and produce infectious virions for longer periods of time with negligible cell death. However, insufficient information in the scientific literature is available which can provide evidence of the establishment of latency in infected macrophages. In patients undergoing HAART very low levels of the population (nearly 0.05%) of macrophages in lymph nodes are infected. Their isolation and characterization for HIV latency is difficult [11]. Of note, Brown and colleagues have developed an *in vitro* model of long termed culture of MDMs infected with recombinant GFP tagged HIV-1. Their results revealed that at least *in vitro,* latency can be established in MDMs [89]. Not surprisingly, since macrophages do not divide, therefore, besides integrated HIV-1 genome, unintegrated and circular forms of virus may also contribute to latency (the pre-integration latency) [90]. However, experimental evidence proving this concept are lacking. Interestingly, human herpesvirus 8 has been shown to reactivate HIV in infected macrophages. This observation raises the possibility of opportunistic infection as one possible mechanism of reactivation in infected macrophages [91].

Furthermore, HIV-1 replication in macrophages is regulated by cytokines and other extracellular stimuli. Based on the stimuli or cytokine profile, macrophages can be polarized into either M1 (classically activated) or M2 (alternatively activated) [92,93]. Cassol and colleagues reported that M1/M2 polarization of MDMs was associated with poor CCR5-dependent HIV-1 infection as compared to non-polarized MDMs. Additionally, in M1 and M2a polarization inhibition may occur at pre-integration or at post-integration steps respectively. Their data indicate that M1/M2a may represent a mechanism of latency induction in macrophages [92].

### 3.3. Latency in CNS Resident Macrophages

Our central nervous system (CNS) is guarded by four types of macrophages. These include microglia, perivascular macrophages, meningeal macrophages and macrophages of the choroid-plexus [94]. Like other tissue macrophages, they are susceptible to HIV-1 infection [55,95,96]. CNS resident macrophages especially microglial cells and perivascular macrophages have special clinical significance as their infection and induction is majorly responsible for the pathogenesis of HIV-1 associated dementia [55,97]. Interestingly, these two types of macrophages have low turnover rates. For example, microglial cells persist for several years in CNS. Therefore, the microglial population represents a potent HIV-1 reservoir in infected patients. The general notion is that microglial cells are infected during early acute phase of infection. Furthermore, in brain tissue of HIV-1 infected patients, more than 10 times the amount of unintegrated viral DNA has been quantitated as compared to integrated DNA [33]. Like other macrophages, microglial cells pose resistance to active HIV-1 infection and host immune response restricts viral growth, therefore pre-integration latency can be also relevant in these cells [55,98,99]. Worth mentioning, during late course of HIV-1 infection when T-cell response is largely hampered coupled with increased proinflammatory stimuli, HIV-1 may reactivate from latency in microglial cells [55,98,99]. Perivascular macrophages have relatively short turnover rate (2–3 months) as compared to microglial cells, thus could be important HIV-1 reservoirs in CNS [25,55].

### 3.4. Latency in Gut-Associated Macrophages

**Figure 2:**
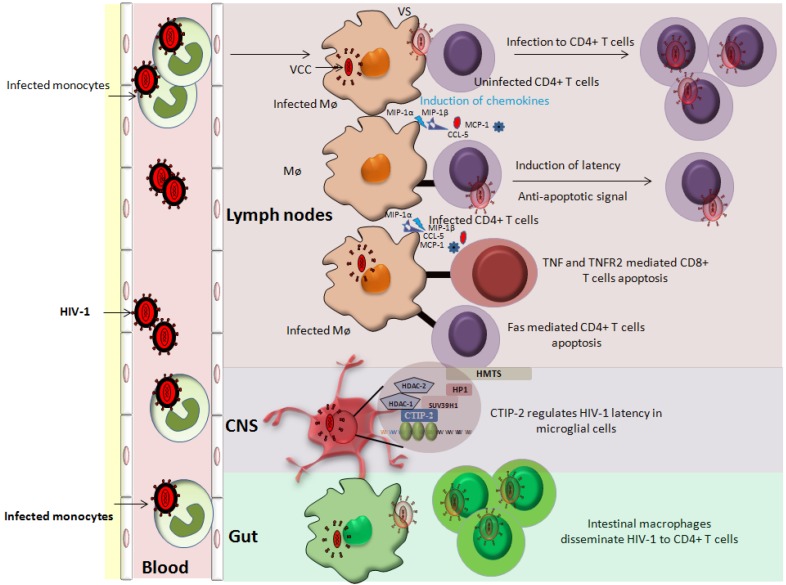
Role of cells of monocyte/macrophage lineage in HIV-1 pathogenesis. Cells of monocyte/macrophage lineage are susceptible to HIV-1 infection. Infected monocytes are an important short-lived viral pool that upon entering tissues differentiated into macrophages. Infected macrophages can transfer virus to the uninfected cells via virological synapses (VS). In addition, infected macrophages release chemokines and cytokines (MIP-1α, MIP-1β, MCP-1 and CCL-5) that attract immune cells (T cells and monocytes) for further infection. Macrophages can induce an anti-apoptotic state in infected CD4^+^ T cells, thereby increasing the viral reservoir. The role of macrophages in inducing latency in CD4^+^ T cells is also postulated. Furthermore, infected macrophages induce the apoptosis of uninfected bystander cells (CD4^+^ and CD8^+^ T cells) further contributing to the HIV-1 pathogenesis. Intestinal macrophages fuel HIV pathogenesis by disseminating the virus to CD4^+^ T cells. Of note, CNS resident macrophages represent an important anatomical viral sanctuary and a difficult target for HIV-1 therapy.

Gut-associated macrophages could play a role in HIV-1 pathogenesis [100]. In the presence of stroma-derived factors, blood monocytes (both uninfected and HIV-1 infected) are differentiated into macrophages of the lamina propria. These infected monocyte-derived intestinal macrophages although low in number however, could represent an important HIV-1 reservoir [101]. Interestingly, intestinal stroma induces the downregulation of CD4 and CCR5 receptors on intestinal macrophages and also display decreased NF-kappa B activation in them [101]. This could be one of the mechanisms of latency in HIV-1 infected intestinal macrophages (Figure 2).

### 3.5. Latency in Dendritic Cells

Dendritic cells (DCs) act as a bridge between the innate and adaptive immune system [86]. DCs have been further classified into three major subtypes which include myeloid DCs (mDCs), Langerhans cells and plasmacytoid DCs (pDCs) [86]. All these subtypes are susceptible to HIV-1 infection [102,103,104,105], however; efficiency of replication is very low. Haase and colleagues reported the presence of 40-fold HIV-1 RNA in follicular dendritic cells as compared to mononuclear immune cells in patients receiving anti-retroviral therapy, suggesting dendritic cells as an important latent reservoir [105]. Furthermore, in a contrasting finding neither proviral DNA nor HIV transcripts have been detected in DCs isolated from HIV patients under the HAART regime [106]. Of note, recently in an elegant report the direct role of mDCs in inducing post integration latency in resting memory CD4^+^ T cells has been shown. In addition, close physical contact between mDCs and resting CD4^+^ T cells has been found to be obligatory in inducing latency in the resting CD4^+^ T cells [107].

## 4. Role of Monocyte/Macrophage Lineage in Regulating Latency in CD4^+^ T Cells

The cells of monocyte/macrophage lineage are key players in HIV pathogenesis. In infected macrophages HIV-1 is present in virus containing compartments (VCCs) [108,109] (Figure 2). These VCCs may provide protective environment since they are not readily accessible by immune effector molecules [108,110]. Besides being a stable viral reservoir, infected MDMs are reported to transmit virus to the uninfected PBLs including CD4^+^ T cells via virological synapses (VS), thereby expanding the size of the viral reservoir [111,112,113] (Figure 2). Since MDMs can transmit HIV-1 to CD4^+^ T cells via physical connection (VS), therefore like mDCs we could speculate that MDMs can also promote latency in CD4^+^ T cells [107].

Recent work from Benaroch’s laboratory revealed that HIV utilizes pre-existing CD36 compartments (VCCs) for its assembly and budding in macrophages. In addition, they demonstrated that application of CD36 specific antibodies results in the inhibition of virus release from VCC. Collectively, their data indicate that anti-CD36 antibodies reduces latent reservoirs by sequestering of the HIV virion within VCCs. As a consequence, virion release from infected macrophages and transmission to CD4^+^ T cells is restricted [114]. Use of anti-CD36 antibodies complement with HAART has been suggested as a potent strategy against HIV residing in infected macrophages [114].

Furthermore, MDMs upon HIV-1 infection secrete several cytokines/chemokines (MIP-1α, MIP-1β, MCP-1 and CCL-5) that attract the lymphocytes in their vicinity (Figure 2) leading to an ideal situation for expanding viral reservoir [115]. Additionally, HIV-1 infected MDMs can induce anti-apoptotic resistance in infected CD4^+^ T cells and promote killing of uninfected bystander cells [116,117] (Figure 2) therefore, promoting the expansion of viral reservoir and latency indirectly [118]. In addition, the role of uninfected macrophages in reactivating HIV-1 in ACH2 and U1 cells has been also reported [119]. Physical interaction of MDMs with ACH2 or U1 cells results in intracellular stimuli that activate NF-kappa B via the release of cytokines from MDMs. These MDM secreted cytokines possibly can reactivate HIV-1 in the target cells [119]. Taken together, it seems to be close a relationship between the cells of monocyte/macrophage lineage in establishing and reactivating latency in CD4^+^ T cells. Moreover, the information derived from the molecular interaction between these two cellular partners can open new avenues for HIV-1 therapeutics.

## 5. Novel Therapeutic Strategies Against HIV-1 Latent Reservoirs

HAART has significantly increased the life span of HIV-1 infected individuals. However, complete eradication of virus is not possible without targeting latent HIV-1 reservoirs, especially in the monocyte/macrophage lineage.

### 5.1. Integrase Inhibitors

There are two kinds of integrase inhibitors employed against HIV-1. They are strand-transfer inhibitors (INSTIs) and 3' processing inhibitors [120,121]. INSTIs binds to the complex formed between viral DNA and integrase resulting in selective 3' end displacement [122]. In addition, INSTIs chelates two Mg^2+^ from the integrase active site [121]. Three INSTIs (raltegravir, dolutegravir and elvitegravirs) have been licensed for clinical use. Similar efficacy of INSTIs has been reported in different reservoirs including CD4^+^ T cells and macrophages [123]. However, in primary human macrophages, INSTIs resistance conferred by a single point mutation has been also reported [124].

Recently, alternative approaches to target the HIV-1 integration step have been tested. For example, lens epithelium-derived growth factor (LEDGF/p75), a co-transactivator of integrase has been targeted by newly designed LEDGINS molecules which interfere with IN-LEDGF/p75 complex and inhibit the integration reaction allosterically [121]. Interestingly, it has been reported that mutations in IN which confer resistance to INSTIs however, are not resistant to LEDGINS [125,126,127]. The use of integrase inhibitors can possibly reduce the chances of latency establishment.

### 5.2. “Flushing out” HIV-1 from the Latent Reservoirs

Once latency is successfully established, INSTIs or LEDGINS are ineffective from that point onwards. According to a hypothesis, latent reservoirs can be targeted in two steps. The first step involved the reactivation followed by their eradication by HAART [128,129]. Theoretically upon reactivation viral genes should be expressed and subsequently the gene product may be processed and present on the cell surface by host cellular machinery. Such cells bearing hallmark of HIV infection should be eliminated by HIV-1 specific cytotoxic T cells [128,129]. In addition, upon reactivation even virus replication can induce cytopathic effects resulting in release of virion progeny [5,128,129]. Ongoing HAART should block the new HIV-1 infection initiated by released viral progeny.

As discussed elsewhere in this review, histone deacetylases (HDACs) play an important role in suppressing HIV-1 proviral DNA expression. HDAC inhibitor (HDACi) for example vorinostat has shown to reactivate HIV-1 from latency in patients undergoing HAART [130]; similar findings have been reported by Elliot and colleagues [131]. However, they did not observe any decline in HIV-1 proviral DNA in CD4^+^ T cells and rectal tissues [128,131].

Most of the clinical data describing the “flushing out” of HIV from latent reservoirs have been described on CD4^+^ T cells. In addition, in most of the clinical trials involving HIV reactivation agents, viral load has been determined specifically in T lymphocytes. Furthermore, sampling of microglial cells from patients is rather a difficult task. There is a scarcity of reports where potency of HIV reactivation agents has been tested in macrophages isolated from patients undergoing clinical trials. Nevertheless, *in vitro* potency of these activators is similar among the cells of monocyte/macrophage lineage and CD4^+^ T cells. For instance, HDACi metacept-1, metacept-3 and oxamflatin have been shown to promote HIV-1 transcription in latently infected monocytic cells and CD4^+^ T cells [132]. Several others HDACis have been found in activating HIV-1 latent reservoirs *in vitro* [128]. In addition to HDACi, several protein kinase C agonists, for example, ingenols, and prostratin have been found effective in activating latent proviral DNA in T cell lines and U1 cells [133,134,135,136]. Similar responses have been reported with histone methyltransferase inhibitors in resting CD4^+^ T cells [56,137,138], NF-kappa B activators [139,140], cytokine therapy [141,142,143] and anti-microRNA inhibitors [144] in CD4^+^ T cells as well as in cells of monocyte/macrophage lineage.

Worth mentioning, findings from Siliciano’s research group demonstrate that although HDACi (SAHA) can reactivate HIV-1 in resting CD4^+^ T cells, it does not however, induce cell death of latently infected cells [145]. Furthermore, they did not observe apoptosis in latently infected resting CD4^+^ T cells even when co-cultured with autologous cytotoxic T cells isolated from HIV-1 patients on HAART. Their findings indicate that activation of cytotoxic T lymphocytes is prerequisite before employing HDACi [145]. These observations might also be applicable for cells of monocyte/macrophage lineage and therefore need extensive investigations. Notably, in chronically infected HIV-1 individuals robust HIV-1 specific cytotoxic response is generally lacking [128,145]. Despite having several tools of reactivating HIV-1 in latent reservoirs, the issue cannot be resolved unless effective anti-HIV-1 cytotoxic response is built in the infected patients. 

### 5.3. Apoptosis Inducing Agents

Alternatively search for pharmacological agents that can induce apoptosis in reactivated cells has begun. The strategy of “Prime, Shock and Kill” [128] has been proposed in which latent reservoirs are made sensitive to apoptosis, followed by reactivation of HIV-1. The priming and shock should ultimately lead to the infected cell death [128]. Several agents including Bcl2 inhibitors, survivin inhibitors and PI3K/AKT inhibitors have potential to induce apoptosis in cancer cells and are proposed to induce similar effect in latent reservoirs [128,146,147,148].

Notably, cells of monocyte/macrophage lineage are more resistant to apoptosis than T lymphocytes. Better understanding of mechanisms of apoptosis in this lineage is critical for the complete elimination of HIV-1 from the infected host. Worth mentioning, in 26L cells (a subclone of U937 cells), the treatment with DNA damaging agents has shown to induce apoptosis. However, in chronically HIV-infected 26L cells or macrophages in which reactivation from latency occurs, anti-apoptotic behavior is observed, indicating the role of viral proteins in inducing this behavior [149]. In another report, persistently infected lymphoid and monocytic cell lines treated with apoptotic inducing agents have been shown to be more resistant as compared to their uninfected counterparts [150].

Of note, macrophage colony stimulating factor (M-CSF) is necessary for the growth and differentiation of monocytes/macrophages [151]. Interestingly, increased expression of M-CSF has been reported in response to HIV-1 infection [152]. In addition, M-CSF is known to regulate apoptosis by inhibiting the expression of TRAIL-1 and upregulating the expression of anti-apoptotic proteins such as Mcl-1 and Bfl-1 [152,153]. Not surprisingly, inhibition of M-CSF receptor activation by the anti-cancer drug imatinib has been reported to enhance the apoptosis in HIV-1 infected macrophages [152]. Besides cellular factors, viral protein Nef [154] and Vpr [155] are known to influence apoptotic behavior of infected cells. Investigations revealed that downregulating the expression of inhibitors of apoptosis (IAPs) in macrophages makes them susceptible to Vpr-induced apoptosis [153]. The possibility of inducing apoptosis in HIV-1 infected macrophages by a cocktail of recombinant Vpr and IAPs inhibitors has been suggested [153]. In addition, altering the expression of M-CSF and downregulating Nef using RNAi could also positively influence apoptosis in infected macrophages [156].

Furthermore, the gene expression profile of apoptotic genes is different between infected U937 cells and CD4^+^ T cells [157]. In addition, circulating monocytes isolated from HIV-1 infected individuals exhibit different program cell death gene signatures as compared to their uninfected counterpart [158]. Therefore, the determination of such signatures can help in specifically targeting cells of monocyte/macrophage lineage infected with HIV-1.

Recently, ciclopirox (an antifungal drug) and deferiprone (an iron chelator) have been reported to induce apoptosis specifically in HIV-1 infected H9 cells and PBMCs [159]. Interestingly, even after cessation of treatment for 12 weeks, no emergence of drug resistant HIV-1 was reported [159]. Such drugs could represent a solution for the complete elimination of proviral DNA from infected reservoirs and coupling such drugs with HAART could help to eradicate HIV-1.

## 6. Conclusions

Decline in HIV-1 related deaths has been associated with early HAART treatment. However, complete cure is not possible without targeting latent viral reservoirs. The cells of monocyte/macrophage lineage are unique in the aspect that they are resistant to the cytopathic effects of virus, have a long lifespan and therefore can disseminate virus for longer periods of time. In addition, they represent anatomical viral sanctuaries where HAART penetration is poor. Several efforts have been made in the direction of reactivating latent reservoirs, enhancing cytotoxic response and triggering apoptosis of infected cells. Combining these developing strategies with established HAART treatment could possibly solve the riddle of a complete cure of HIV-1 by targeting both infected T-cells and cells of the monocyte/macrophage lineage.
